# Fibroblasts Mediate Ectopic Bone Formation of Calcium Phosphate Ceramics

**DOI:** 10.3390/ma15072569

**Published:** 2022-03-31

**Authors:** Liangliang Fu, Qin Zhao, Jiaojiao Li, Zifan Zhao, Min Wang, Huifang Sun, Haibin Xia

**Affiliations:** 1 The State Key Laboratory Breeding Base of Basic Science of Stomatology (Hubei-MOST) & Key Laboratory of Oral Biomedicine Ministry of Education, School & Hospital of Stomatology, Wuhan University, Wuhan 430079, China; fuliangliang@whu.edu.cn (L.F.); zhaoqin@whu.edu.cn (Q.Z.); 2021203040020@whu.edu.cn (J.L.); zzfan1020@163.com (Z.Z.); 83wangmin@whu.edu.cn (M.W.); huifang.sun@whu.edu.cn (H.S.); 2 Department of Oral Implantology, School and Hospital of Stomatology, Wuhan University, Wuhan 430079, China

**Keywords:** bone regeneration, calcium phosphate ceramics, fibroblast

## Abstract

Heterogeneity of fibroblasts directly affects the outcome of tissue regeneration; however, whether bioactive ceramics regulate bone regeneration through fibroblasts is unclear. Ectopic bone formation model with biphasic calcium phosphate (BCP) implantation was used to investigate the temporal and spatial distribution of fibroblasts around ceramics. The effect of BCP on L929 fibroblasts was evaluated by EdU assay, transwell assay, and qRT-PCR. Further, the effect of its conditioned medium on osteogenic differentiation of bone marrow mesenchymal stem cells (BMSCs) was confirmed by ALP staining. SEM and XRD results showed that BCP contained abundant micro- and macro-pores and consisted of hydrogen-apatite (HA) and β-tricalcium phosphate (β-TCP) phases. Subsequently, BCP implanted into mice muscle successfully induced osteoblasts and bone formation. Fibroblasts labelled by vimentin gathered around BCP at 7 days and peaked at 14 days post implantation. In vitro, BCP inhibited proliferation of L929 fibroblast but promoted its migration. Moreover, expression of *Col1a1*, *Bmp2*, and *Igf1* in L929 treated by BCP increased significantly while expression of *Tgfb1* and *Acta* did not change. ALP staining further showed conditioned media from L929 fibroblasts treated by BCP could enhance osteogenic differentiation of BMSCs. In conclusion, fibroblasts mediate ectopic bone formation of calcium phosphate ceramics.

## 1. Introduction

In the field of bone engineering, ideal bone substitutes, such as bioactive ceramics, are designed to enhance stem cell recruitment, attachment, and osteogenic differentiation [1]. In addition to the direct effects on stem cell, bone substitutes are assumed to create pro-regenerative niche to support stem cell proliferation and osteogenic differentiation [2]. Hence, it is important to uncover the interaction between bone substitutes and the surrounding niche after inner implantation.

Ectopic bone formation model is widely used to investigate the interaction between bone substitutes and surrounding niche during bone formation [3,4]. Biphasic calcium phosphate (BCP) ceramics have been proved to promote ectopic bone formation after subcutaneous or muscle pouch implantation in rodents or larger animals (dog, goat, and sheep) [5]. In this model, BCP ceramics have excellent biocompatibility and osteo-inductive ability [6,7]. In addition, BCP has been reported to regulate the phenotype of macrophage, dendric cell, and T cell to recruit bone marrow mesenchymal stem cells (BMSCs) and enhance osteogenic differentiation of BMSCs [2,8].

Besides immune cells, fibroblast is another important player in bone regenerative niche remodeling [9,10,11]. Fibroblasts can respond to different milieus with distinct phenotypes, including pro-regenerative phenotype and pro-fibrotic phenotype, leading to different outcomes of tissue regeneration [12,13]. A growing body of studies revealed that the phenotype of fibroblasts could be modulated to enhance tissue regeneration [14,15]. Previous studies prepared a series of bioactive hydrogel scaffolds to regulate fibroblast migration, collagen synthesis, and cytokine secretion to modify surrounding niche and promote skin regeneration [14,16]. In addition, fibroblasts activated by hydrogel enhanced angiogenesis [17]. However, the role of fibroblasts during BCP induced bone formation remains unknown.

Here, in order to decipher the interaction among fibroblasts, BCP, and bone formation, we proposed hypothesis that pro-regenerative phenotype of fibroblasts was involved in bone formation induced by bioactive BCP. Firstly, we successfully prepared and used bioactive BCP to induce bone formation in ectopic bone formation model. And HE and IHC staining revealed that abundant fibroblasts gathered around ceramics at 7 days post implantation. In vitro, BCP ceramics decreased proliferation of L929 fibroblasts but promoted its migration. qRT-PCR results have shown that *Col1a1* expression was increased but *Tgfb1* and *Acta* (encoding α-SMA) expression was not changed. Interestingly, osteogenic factors including *Bmp2* and *Igf1* in L929 fibroblasts with BCP were upregulated at mRNA level. ALP staining further confirmed that BCP promoted osteogenic differentiation of BMSCs through fibroblasts. Our results showed fibroblasts were involved in bone formation and bioactive ceramics regulated the phenotype of fibroblasts to promote osteogenic differentiation of BMSCs. Furthermore, our study provided a new strategy of bone substitutes design for bone regeneration.

## 2. Materials and Methods

### 2.1. Preparation of BCP Ceramics

BCP ceramics were synthesized using the chemical precipitation method as previously described [18]. BCP ceramics consisted of 60% hydroxyapatite (HA) and 40% β-tricalcium phosphate (β-TCP). After hydrogen peroxide foaming, the ceramics were mixed with 0.5 wt% methyl cellulose and sintered at 1100 °C for 3 h. Finally, uniform size of ceramics was obtained by grounding and sieving before further study.

### 2.2. Morphological and Phase Analysis of BCP

For the morphological analysis, the gold coating under vacuum (Quorum Technologies, Lewes, UK) was conducted on the membrane surface for 5 min prior to capturing the image. Then, the surface of BCP ceramics was assessed by scanning electron microscope (SEM, Hitachi, Tokyo, Japan). The images were taken at an accelerating voltage of 5.0 kV. Phase analysis of the prepared BCP ceramics was performed by X-ray diffractometer (Malvern Panalytical, Malvern, UK). In detail, crystalline phase analysis was operated with a Cu Kα radiation with 1.5406 Å wavelength generated at 40 kV and 40 mA. The diffraction angles (2θ) of each sample were measured from 20° to 60° at a scan speed of 2°/min and a step size of 0.03°. The image of XRD was proceeded with OriginPro 2019 (OriginLab, Northampton, MA, USA).

### 2.3. Mice and Experimental Model

Animal experiments were conducted according to the guidelines of the Declaration of Helsinki and were approved by the Ethics Committee of the Wuhan University (registration no. 2020-A30). Six to eight-week-old female C57BL/6 mice (20–25 g in weight) from Hubei Provincial Center for Disease Control and Prevention (Wuhan, China) were raised in SPF animal laboratory of Wuhan university and used for ectopic bone formation model and BMSCs isolation. In this study, 15 C57BL/J mice were used in ectopic bone formation models and divided into 5 groups for each time points (1d, 3d, 7d, 14d, and 28d post ceramics implantation). Mice were given sodium pentobarbital for pain relief before surgery and sacrifice. For ectopic bone formation model, a tiny incision about 10 mm in length and 5 mm in depth was made along with the gastrocnemius muscle, and then 5 BCP ceramic particles with 0.5–0.8 mm diameter were gently implanted into the incision. Muscle and skin were sutured tightly with 5–0 line. At indicated times, mice were euthanized by carbon dioxide treatment before sample harvest.

### 2.4. Histological Staining

The harvested samples were used for Haematoxylin-eosin (H&E), Masson staining, and immunohistochemical (IHC) staining. Initially, the samples were fixed by 4% paraformaldehyde and then immersed in 15% EDTA for two weeks. Next, the decalcified samples were dehydrated, followed by gradient ethanol, and embedding in paraffin. For HE, slices were stained by haematoxylin and eosin, respectively. Masson staining was performed following the manufacturer’s protocol (MXB biotechnologies, Fuzhou, China). IHC staining was performed according to the manufacture’s protocol (MXB Biotechnologies, Fuzhou, China) and was visualized by 3,3-diaminobenzidine tetrahydrochloride (Zhongshan Biotechnology, Zhongshan, China). The anti-vimentin antibody used in the IHC staining was diluted at 1:200 (ABclonal, Wuhan, China). Images of HE and IHC staining were taken with optical microscope (Olympus, Tokyo, Japan).

### 2.5. Cell Culture

L929, a fibroblast cell line, was obtained from China center for type culture collection and maintained in Dulbecco’s Modified Eagle’s Medium (DMEM, Gibco, Waltham, MA, USA) containing 10% fetal bovine serum (FBS, Gibco, Waltham, MA, USA) under humid conditions with 5% CO_2_. When L929 fibroblasts reached about 60–70% confluency in 10 cm dish with 6 mL complete culture media, about 0.5 mg BCP ceramics particles were added to stimulate L929. After 24 h, conditioned culture media was collected followed by centrifugation at 1000 rpm. The supernatant was used for osteo-induction of BMSCs.

Bone marrow mesenchymal stem cells (BMSCs) were isolated as previously described [18]. Briefly, bone marrow was flushed from the femoral bone of C57BL/6 mice. Next, the cells were cultured with α-Modified Eagle’s Medium (α-MEM, Gibco, Waltham, MA, USA) supplemented with 20% FBS, and the culture medium was refreshed every three days. After three passages, BMSCs were used in osteogenic differentiation [19].

### 2.6. Cell Proliferation Assay

To evaluate the proliferation ability of L929 fibroblasts treated by BCP particles, EdU cell proliferation staining was performed using an EdU kit (Beyotime, Shanghai, China). Briefly, L929 cells (2 × 10^5^ cells/well) were seeded in 12-well plates and cultured with 5 BCP particles for 24 h. Subsequently, cells were incubated with EdU for 3 h, fixed with 4% paraformaldehyde for 10 min, and permeated with 0.3% Triton X-100 for 15 min. Then, the cells were incubated with the Click Reaction Mixture for 30 min in a dark place and also incubated with Hoechst 33342 for 10 min. Images of EdU staining were acquired by fluorescent microscope (Zeiss, Oberkochen, Germany). Semi-quantitative analysis was conducted based on the ratio of EdU positive cells. Experiments were repeated three times independently. From each experiment image, three randomized regions of interest were used to calculate the ratio of EdU positive cells. For each group, maximal and minimal data were excluded and the remaining data were used for statistical analysis.

### 2.7. Transwell Assay

L929 cells were seeded in 24-well upper chamber (5 × 10^3^ cells per chamber) and 2–3 BCP particles were added to the lower chambers. After 24 h stimulation, cells were fixed in 4% formaldehyde for 10 min and stained with crystal violet (Beyotime, Shanghai, China). After washing with PBS thrice, the chambers were taken images by microscope (Olympus, Tokyo, Japan). Semi-quantitative analysis was conducted based on the number of cells stained by crystal violet. Three experiments were repeated independently. For each experiment, three randomized regions of interest were sectioned from each image to calculate the number of cells stained by crystal violet. For each group, maximal and minimal data were excluded and the remaining data were used for statistical analysis.

### 2.8. Quantitative Real—Time PCR

L929 cells (5 × 10^5^ cells/well) were seeded in 6-well plates and cultured with 5 BCP particles for 24 h. Then, total RNA of L929 fibroblasts was isolated with Trizol reagent (Invitrogen, Waltham, MA, USA) [20]. The ratio of absorbance at 260 nm and 230 nm measured by Nanodrop2000 (Thermo Fisher Scientific, Waltham, MA, USA) must be above 2.0. Complementary DNA was synthesized with 1 μg of total RNA and was carried out according to PrimeScript RT--PCR Kit (TaKaRa Bio, Shiga, Japan). The gene expression was calculated in ΔΔCT method and was normalized to *Gapdh*. The primer sequences of target genes were listed in Supplementary Table S1.

### 2.9. Alkaline Phosphatase (ALP) Staining

Osteo-induction medium (OS) consisted of α-MEM supplemented with 10% FBS, 10 mmol/L β-glycerophosphate, 10 nM dexamethasone, and 50 μg/mL ascorbic acid (Sigma, St. Louis, MS, USA) [2]. BMSCs (2 × 10^5^ cells/well) were seeded in 12-well plates. When nearly 80% cell confluency was reached, cells were maintained with OS alone or OS supplemented with 5 BCP particles (OS + BCP) or OS supplemented with 20% conditioned media from L929 treated by BCP particles (OS + BCP + L929). Osteo-induction medium was replaced every 2 days [21]. ALP staining was carried out after 7-day osteo-induction. Cells were fixed and then stained in accordance with the manufacture’s protocol (Beyotime, Shanghai, China). Finally, micro-structures of ALP staining were taken by optical microscope (Olympus, Tokyo, Japan). Semi-quantitative analysis was conducted based on the percentage of area of cells stained by ALP. Three randomized regions of interest were captured from each group. The area of cells stained by ALP was calculated with Image J v2.0 (NIH, Bethesda, MD, USA).

### 2.10. Statistical Analysis

The data were shown as the mean ± standard deviation (SD). Cell proliferation assay and transwell assay were analyzed using *n* = 7 replicates. qRT-PCR and ALP staining used *n* = 3 replicates. Statistical significance was analyzed with the GraphPad Prism software 8.0 (GraphPad Software, San Diego, CA, USA) and *p* < 0.05 were considered to be statistically significant. Multiple comparison in ALP staining was conducted by the Tukey post-hoc test using a one-way analysis of variances (ANOVA). Student’s *t* test was used for two-group comparison.

## 3. Results

### 3.1. Bioactive BCP Implantation in Muscle Leads to Bone-like Formation

**Figure 1:**
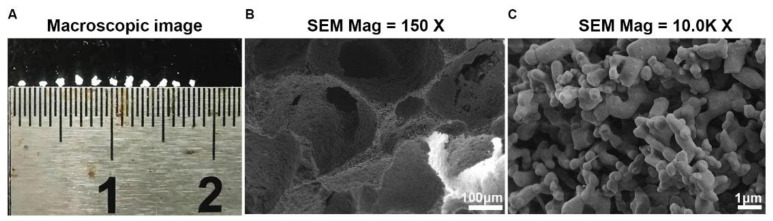
Morphological characteristics of BCP ceramics. (**A**) Macroscopic image showed the sizes of BCP particles ranged from 0.5–0.8 mm. (**B**,**C**) SEM image showed the macro- and micro-pores ranged from 20–400 μm and 0.3–1.8 μm, respectively. Scale bar: 100 μm and 1 μm, respectively.

**Figure 2:**
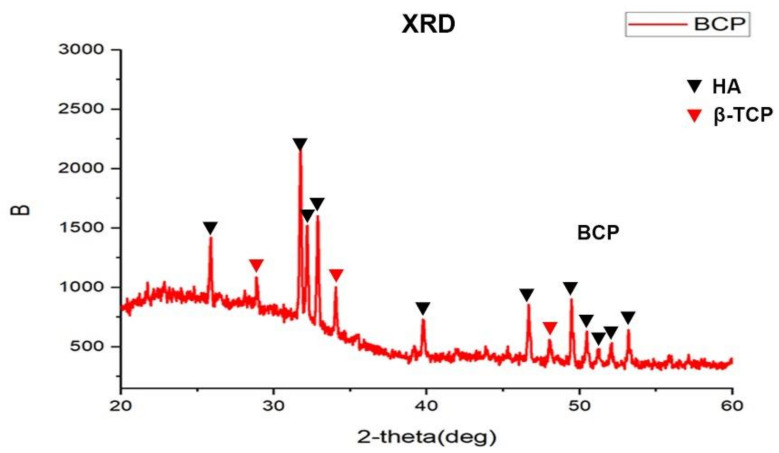
X-ray diffraction pattern of BCP ceramics. The pattern for BCP ceramics has several HA and β-TCP peaks.

**Figure 3:**
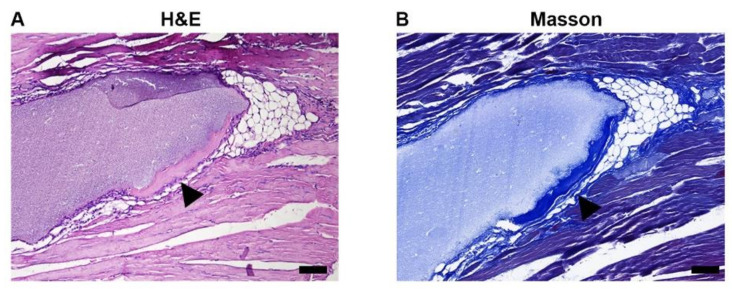
Bone-like formation induced by BCP ceramics. (**A**) HE staining of BCP in muscle after 4-week implantation showed bone-like structure formed around BCP ceramics and even some osteoblasts were found. (**B**) Masson staining of BCP in muscle after 4-week implantation showed bone-like structure formed as indicated by black triangles. Scale bar: 20 μm.

To synthetize the osteo-inductive CaP ceramics, porous BCP was prepared with chemistry precipitation method and particle sizes of BCP were limited to 0.5–0.8 mm (Figure 1A). The SEM image showed macro- or micro- pores of BCP particle ranged from 20–400 μm and 0.3–1.8 μm, respectively (Figure 1B,C). X-ray diffraction (XRD) analysis of BCP ceramics further confirmed that it consisted of a mixture of hydroxyapatite (HA) and β-tricalcium phosphate (β-TCP) phases (Figure 2). In line with previous study, XRD analysis in our study showed that the material had the characteristic diffraction peaks of HA at 25.9°, 31.8°, 32.3°, 33.0°, 39.8°, 46.7°, 49.5°, 50.5°, 51.3°, 52.1°, and 53.0°, and diffraction peaks of β-TCP at 28.9°, 34.0°, and 47.6° [22]. To investigate the osteo-inductive ability of synthetized BCP ceramics, BCP ceramics were implanted into gastrocnemius muscle of mice. After 4-week implantation, BCP ceramics and surrounding tissue were collected and prepared for histological assessments. HE and Masson staining showed that bone-like structure was formed around BCP ceramics (Figure 3A,B). Overall, we successfully synthetized osteo-inductive BCP ceramics.

### 3.2. Temporal and Spatial Distribution of Fibroblasts around BCP during Bone-like Formation

**Figure 4:**
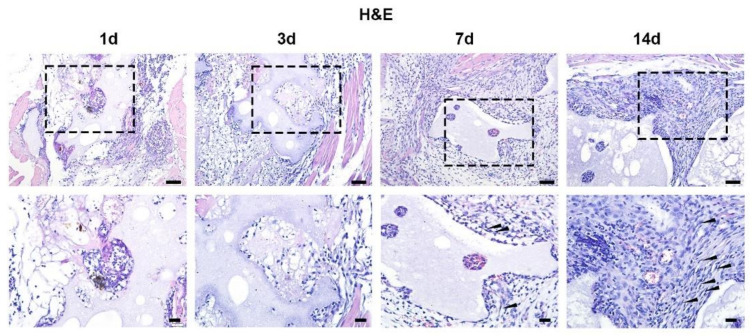
Biological reactions induced by BCP ceramics. Consecutive HE staining of BCP after implantation in muscle showed abundant immune cells infiltrated around BCP ceramics at day 3 after implantation and functional cells like fibroblasts emerged after 7-day implantation. Fibroblasts which are featured with spindle shape gathered around BCP ceramics at day 14 after implantation as indicated by black triangles in magnified images. Scale bar: 50 μm and 20 μm, respectively.

**Figure 5:**
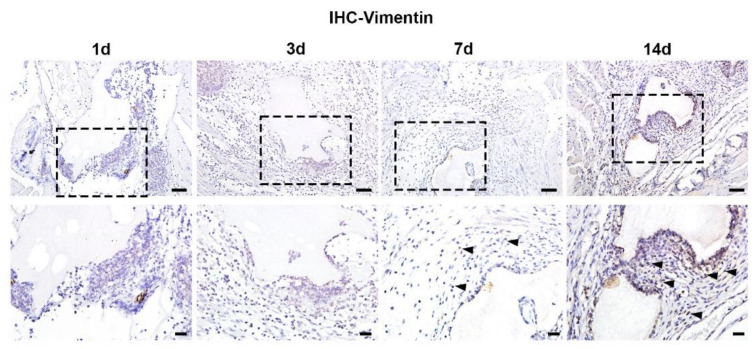
Temporal and spatial distribution of fibroblasts around BCP. Consecutive immunohistochemical staining of BCP after implantation in muscle showed the number of fibroblasts labelled by vimentin increased dramatically during 14-day implantation as indicated by black triangles in magnified images. Scale bar: 50 μm and 20 μm, respectively.

To evaluate the dynamic changes during bone-like formation induced by BCP ceramics, different time points of samples from day 1 to day 14 after implantation were collected and prepared for histological observations. HE staining shows abundant infiltration of immune cells around BCP at day 3 after implantation. During day 7 to day 14, functional cells including epithelial cells and fibroblasts emerged and dramatically increased (Figure 4). To further investigate the temporal and spatial distribution of fibroblasts during bone formation, fibroblasts were labelled by anti-vimentin antibody with immunohistochemical staining. It is interesting that few vimentin-positive fibroblasts were present at day 3, while a large number of vimentin-positive fibroblasts emerged and gathered around BCP ceramics at day 7 and day 14 (Figure 5).

### 3.3. BCP Promoted Migration and Collagen Secretion of Fibroblasts

**Figure 6:**
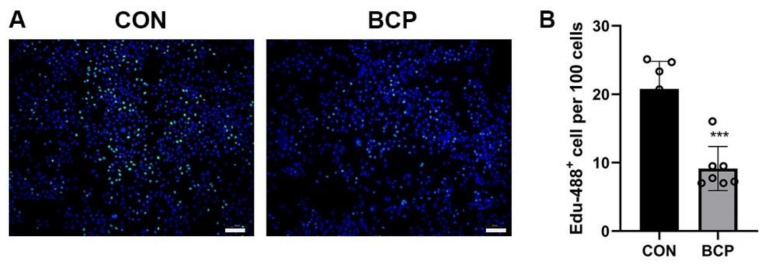
Proliferation ability of L929 fibroblasts cultured with BCP particles. (**A**) Representative images of Edu staining and (**B**) Semi-quantitative results showed proliferation of L929 fibroblasts was inhibited by BCP ceramics and the number of proliferative fibroblasts labelled by green fluorescence in BCP group was reduced to half of that in control group. Scale bar: 120 μm. *** indicated *p* < 0.001.

**Figure 7:**
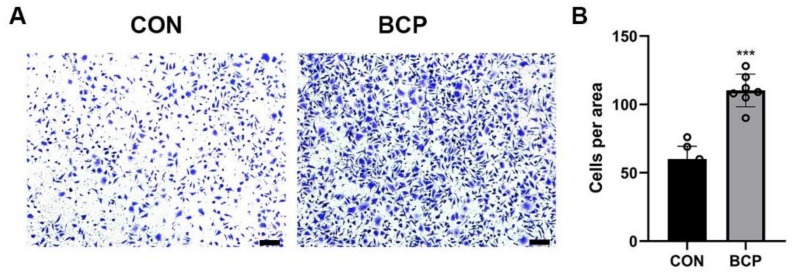
Migration ability of L929 fibroblasts induced by BCP ceramics. (**A**) Representative images of transwell assay and (**B**) Semi-quantitative results showed migration ability of L929 fibroblasts was elevated by BCP ceramics. Scale bar: 100 μm. *** indicated *p* < 0.001.

**Figure 8:**
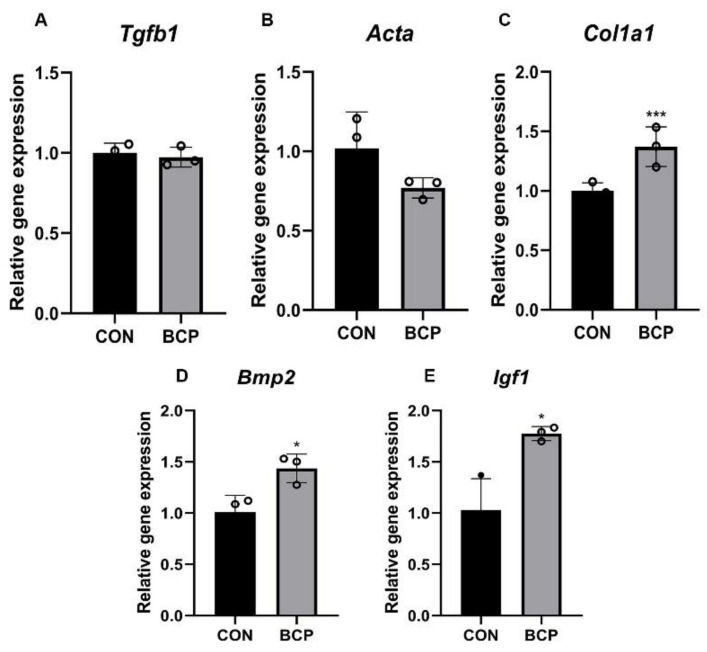
Gene expression of L929 fibroblasts induced by BCP ceramics. (**A**–**C**) *Tgfb*1, *Acta*, *Col1a1*; and (**D**,**E**) *Bmp2* and *Igf1* expression in fibroblasts treated by BCP ceramics were evaluated by qRT-PCR. Relative gene expression was normalized by *Gapdh*. *** indicated *p* < 0.001; * indicated *p* < 0.05.

To investigate the possible effect of BCP particles on fibroblasts, L929 fibroblasts were cultured with BCP particles for 24 h. In terms of proliferation, EdU assay showed the number of proliferative fibroblasts labelled by green fluorescence were significantly decreased after addition of BCP particles (Figure 6A,B). Interestingly, transwell assay showed the migration ability of fibroblasts were enhanced significantly by BCP ceramics (Figure 7A,B). Furthermore, *Col1a1* gene, a collagen marker, was significantly activated while *Tgfb1* and *Acta* (encoding α-SMA) expression was not altered. (Figure 8A–C). Notably, osteogenic growth factors such as *Bmp2* and *Igf1* expression was significantly increased in fibroblasts induced by BCP ceramics.

### 3.4. BCP Enhanced the Mineralization of the BMSCs through Fibroblasts

**Figure 9:**
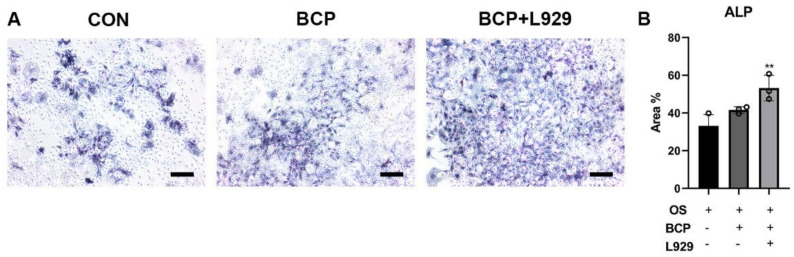
BCP ceramics promoted BMSCs osteogenic differentiation through fibroblasts. (**A**) Representative images and (**B**) semi-quantitative results of ALP staining of BMSCs after 7-day induction showed BMSCs cultured with conditioned media from L929 and BCP were more likely to differentiate into osteoblasts. Scale bar: 100 μm. ** indicated *p* < 0.01.

To uncover the role of fibroblasts in bone-like formation induced by BCP ceramics, a co-culture model of BMSCs and fibroblasts was established. Initially, we collected the supernatant of the L929 fibroblasts cultured with BCP particles. Subsequently, BMSCs were induced with osteogenic induction media (OS) alone or OS supplemented with BCP particles (OS + BCP) or OS supplemented with conditioned media from L929 treated by BCP particles (OS + BCP + L929). After 7-day induction, ALP staining showed that BCP particles could not regulate BMSCs osteogenic differentiation. However, supernatant of fibroblasts with BCP particles could significantly promote osteogenic differentiation of BMSCs (Figure 9A). The percentage of area of osteo-like cells stained by ALP in OS + BCP + L929 group was two times as high as that in OS group (Figure 9B).

## 4. Discussion

Calcium phosphate ceramics received a great deal of interest, especially for the hard tissue repair and regeneration applications due to their biocompatibility, osteo-conductivity, and biodegradability [23]. In this study, we prepared BCP as previously described [24] and used a model of intramuscular implantation in mice to confirm the osteo-inductive capability of BCP. Intramuscular implantation was simple and widely used, which allow for relatively controlled bone formation in in vivo experiment. In this study, we found bone-like formation after 4-week BCP implantation in murine skeletal muscle, which was consistent with previous studies [25,26]. In larger animals, 8- or 12-week follow ups were normally conducted. van Gaalen et al. found around 21% bone formation was around materials in goats after 12-week follow up [27]. Barbieri D et al. also found around 20% bone formation existed after 12-week implantation in sheep dorsal muscle [28]. With long-term implantation, more bone formation could be formed. Therefore, longer follow ups for BCP implantation in murine skeletal muscle are needed in the future studies.

Our study, for the first time, showed that non-fibrotic fibroblasts were formed around BCP ceramics in ectopic bone formation model. In vitro study, BCP particles enhanced the migration ability of L929 fibroblasts and collagen synthesis. *Tgfb1* and *Acta* (encoding α-SMA), typical pro-fibrosis markers, in L929 fibroblasts cultured with BCP were not activated, which indicated that fibroblasts induced by BCP showed non-fibrotic features. Moreover, expression of pro-regenerative factors such as *Bmp2* and *Igf1* was significantly increased. ALP staining further confirmed that these fibroblasts after BCP treatment effectively promoted BMSCs osteogenic differentiation. Our study indicated pro-regenerative phenotype of fibroblasts induced by BCP ceramics contributed to bone formation. Furthermore, our study provides new strategy for biomaterials designs in bone regeneration.

It is well known that surface topography and porosity are critical factors for osteo-inductive capability of BCP. In this study, SEM images of BCP showed abundant macro- and micro pores were present in ceramic. According to SEM image, the micro-pores diameter ranged from 300–1800 nm. In the XRD, we attempted to apply the Debye–Scherrer equation to obtain the size of BCP crystallite. As for HA phase at 26°, 32°, 40 °, and 50°, the estimated crystallite size ranged from 30.7–224.5 nm. As for β-TCP phase at 28 °and 34°, the crystallite size ranged from 261.50–969.4 nm. According to previous studies, the Debye–Scherrer equation was normally used to evaluate nano materials, the size of which was below 100 nm [29]. When crystallite size was above 100 nm, the estimated size obtained by the Debye–Scherrer equation was lower than the size estimated by SEM. Moreover, the SEM of BCP showed a semi-spherical shape with a high degree of aggregation, which might also account for larger crystallite size than estimated results by XRD. Macropore sizes effectively affected the resorption of ceramic scaffolds and the bone regeneration. For example, the intermediate macropore size (20–400 μm) of ceramic was associated with low resorption and excellent bone formation [30]. Low resorption of ceramic scaffold could release sustained calcium ion and trigger CaSR signal pathway [31]. This may explain, at least in part, the osteo-inductive capability of the BCP synthesized in this study.

In our study, fibroblasts labelled by vimentin marker evidently gathered around BCP materials 7 days after implantation and L929 fibroblasts induced by BCP in vitro showed low fibrotic potentials. However, the contribution of these fibroblasts in bone formation was largely overlooked. Fibroblasts were historically considered to unavoidably lead to fibrosis in bone regeneration. In fact, fibroblast had extensive heterogeneity and had programmable potentials. According to transcript level and anatomic position, fibroblasts in muscle could be divided into perimysial cells, paramysial cells, fibro/adipogenic progenitors, perivascular fibroblasts, and endomysial fibroblasts [12,32]. Fibroblasts could also be divided into pro-fibrotic and pro-regenerative phenotypes according to outcomes of regeneration [13,33]. Pro-fibrotic fibroblasts tended to express high expression level of collagen protein encoding genes such as *Col1a1*, *Col3a1*, and myo-fibroblast differentiation markers such as *Poatn*, *Acta2*, and *Pdgfra*. Pro-regenerative fibroblasts had high expression level of metalloproteinase genes such as *Mmp1*, *Mmp3*, and *Mmp10*, which degrade collagen and reduce fibrosis [34]. Moreover, pro-regenerative fibroblasts, which showed high expression level of *Mfge8*, could enhance secretion of VEGF and increase angiogenesis [35]. Overall, fibroblasts have extensive heterogeneity and phenotype of fibroblasts modulated by BCP ceramics was likely to be non-fibrotic.

In our study, BCP increased expression level of growth factors in fibroblasts and the supernatant from theses fibroblasts effectively promoted BMSCs osteogenic differentiation. These interesting results indicated a programable phenotype of fibroblast could be induced by BCP into pro-regenerative phenotype. However, the mechanism between fibroblasts and stem cells osteogenic differentiation was unclear. Previous studies have showed that fibroblasts were not only involved in extracellular matrix homeostasis and remodeling, but also secreted a plethora of growth factors to regulate surrounding cells. For example, fibroblasts have been reported to secret TGF-β, BMP2, VEGF, and IGF1 to modulate stem cells [9]. In line with previous studies, our study also revealed increased expression of *Bmp2* and *Ifg1* in fibroblasts cultured with BCP.

In addition, some fibroblasts have self-renewal and multipotent ability. Fibroblasts have been reported to differentiate into lipocyte, endothelial cells, chondrocytes, and osteoblasts [36,37]. An interesting phenomenon of bone-like formation in soft tissue might enlighten us [38]. Ectopic bone formation was a pathological phenomenon and PDGFRα+ cell, a typical fibroblast, was the main cause of bone-like formation in soft tissue [39,40,41]. PDGFRα+ cells, tissue-resident fibro/adipogenic progenitor cells, differentiate into osteoblasts responding to inflammatory reactions after severe injury [11,42]. Therefore, whether fibroblasts induced by BCP ceramics has the potential to transit into osteogenic cells should be further investigated.

## 5. Conclusions

In this study, bioactive BCP ceramics with abundant macro- and micro-pores was prepared by mixing HA and β-TCP, which successfully induced ectopic bone formation after four-week implantation in murine skeletal muscle. During the process of bone-like formation induced by BCP, fibroblasts labelled by vimentin marker gathered around materials after 7-day implantation and peaked at day 14, which demonstrated fibroblasts were possibly involved in bone-like formation. Furthermore, in in vitro study, BCP particles enhanced the migration ability of L929 fibroblasts and collagen synthesis. *Tgfb1* and *Acta* (encoding α-SMA), typical pro-fibrosis markers, in L929 fibroblasts cultured with BCP were not activated, which indicated that fibroblasts induced by BCP showed non-fibrotic features. In addition, we found BCP could promote expression of growth factors such as *Bmp2* and *Igf1* in L929 fibroblasts. In addition, ALP staining indicated that L929 pre-treated by BCP particles could effectively enhance osteogenic differentiation of BMSCs than BCP alone. Overall, we concluded that fibroblasts mediate ectopic bone formation of calcium phosphate ceramics.

## Data Availability

The data presented in this study are available on request from the corresponding author.

## Supplementary Materials

The following supporting information can be downloaded at https://www.mdpi.com/article/10.3390/ma15072569/s1, Table S1: Gene sequences for qRT-PCR.

## Abbreviations

SEM	Scanning Electron Microscope
XRD	X-ray Diffraction
BCP	Biphasic Calcium Phosphate
BMSC	Bone Marrow-Derived Mesenchymal Stem Cells
CaP	Calcium Phosphates
ALP	Alkaline phosphatase
IHC	Immunohistochemistry
HE	Haematoxylin-eosin
HA	Hydroxyapatite
TCP	Tricalcium Phosphate
