# Supplementary material for: Fibroblasts Mediate Ectopic Bone Formation of Calcium Phosphate Ceramics
Source: Materials (Basel). 2022 Mar 31;15(7):2569. doi: 10.3390/ma15072569 (PMC9000332; doi:10.3390/ma15072569)
Supplement: Supplementary file 1 [file materials-15-02569-s001.zip › materials-1616919-supplementary.pdf]

materials

Supplementary Materials

Fibroblasts Mediate Ectopic Bone Formation of Calcium Phosphate Ceramics

Liangliang Fu [1,2,†], Qin Zhao [1,†], Jiaojiao Li [1], Zifan Zhao [1], Min Wang [1,2], Huifang Sun [1,2] and Haibin Xia [1,2,*]

[1] The State Key Laboratory Breeding Base of Basic Science of Stomatology (Hubei-MOST) & Key Laboratory of Oral Biomedicine Ministry of Education, School & Hospital of Stomatology, Wuhan University, Wuhan, 430079, China; fuliangliang@whu.edu.cn (L.F.); zhaoqin@whu.edu.cn (Q.Z.); 2021203040020@whu.edu.cn (J.L.); zzfan1020@163.com (Z.Z.); 83wangmin@whu.edu.cn (M.W.); huifang.sun@whu.edu.cn (H.S.)

[2] Department of Oral Implantology, School and Hospital of Stomatology, Wuhan University, Wuhan, 430079, China

* Correspondence: xhaibin@whu.edu.cn

† Liangliang Fu and Qin Zhao contributed equally to this work.

Table S1. Gene sequences for qRT-PCR.

Genes	Primer sequence
Col1a1 F	TGGCAAAGACGGACTCAAC
Col1a1 R	GGCAGGAAGCTGAAGTCATAA
Tgfb1 F	TGATACGCCTGAGTGGCTGTCT
Tgfb1 R	CACAAGAGCAGTGAGCGCTGAA
Acta2 F	TGCTGACAGAGGCACCACTGAA
Acta2 R	CAGTTGTACGTCCAGAGGCATAG
Bmp2 F	AACACCGTGCGCAGCTTCCATC
Bmp2 R	CGGAAGATCTGGAGTTCTGCAG
Igf1 F	CATACTGCTTCCTTGGGGTCA
Igf1 R	CCAGGCAAGGGGAATGATCT
Gapdh F	AGGTCGGTGTGAACGGATTTG
Gapdh R	GGGGTCGTTGATGGCAACA
